# Serum deprivation limits loss and promotes recovery of tenogenic phenotype in tendon cell culture systems

**DOI:** 10.1002/jor.24761

**Published:** 2020-06-10

**Authors:** Marc van Vijven, Stefania L. Wunderli, Keita Ito, Jess G. Snedeker, Jasper Foolen

**Affiliations:** ^1^ Orthopaedic Biomechanics, Department of Biomedical Engineering Eindhoven University of Technology Eindhoven The Netherlands; ^2^ Institute for Complex Molecular Systems Eindhoven University of Technology Eindhoven The Netherlands; ^3^ Orthopaedic Biomechanics Laboratory University Hospital Balgrist, University of Zurich Zürich Switzerland; ^4^ Institute for Biomechanics, ETH Zurich Zürich Switzerland

**Keywords:** fetal bovine serum, gene expression, murine tendon models, phenotypic drift, tendon(‐derived) cells

## Abstract

Current knowledge gaps on tendon tissue healing can partly be ascribed to the limited availability of physiologically relevant culture models. An unnatural extracellular matrix, high serum levels and random cell morphology in vitro mimic strong vascularization and lost cell elongation in pathology, and discord with a healthy, in vivo cell microenvironment. The thereby induced phenotypic drift in tendon‐derived cells (TDCs) compromises the validity of the research model. Therefore, this research quantified the extracellular matrix (ECM)‐, serum‐, and cell morphology‐guided phenotypic changes in tendon cells of whole tendon fascicle explants with intact ECM and TDCs cultured in a controlled microenvironmental niche. Explanted murine tail tendon fascicles were cultured in serum‐rich or serum‐free medium and phenotype was assessed using transcriptome analysis. Next, phenotypic marker gene expression was measured in in vitro expanded murine tail TDCs upon culture in serum‐rich or serum‐free medium on aligned or random collagen I patterns. Freshly isolated fascicles or TDCs served as native controls. In both systems, the majority of tendon‐specific genes were similarly attenuated in serum‐rich culture. Strikingly, 1‐week serum‐deprived culture—independent of cell morphology—converged TDC gene expression toward native levels. This study reveals a dynamic serum‐responsive tendon cell phenotype. Extracting fascicles or TDCs from their native environment causes large changes in cellular phenotype, which can be limited and even reversed by serum deprivation. We conclude that serum‐derived factors override matrix‐integrity and cell morphology cues and that serum‐deprivation stimulates a more physiological microenvironment for in vitro studies.

## 1 INTRODUCTION

Healthy tendon tissue is poorly vascularized,^1^, ^2^, ^3^ and characterized by a network of elongated fibroblasts, named tenocytes, embedded in an anisotropic extracellular matrix (ECM) mainly composed of collagen I.^4^ In response to physiological loading, tenocytes remodel the tissue based on functional mechanical demands. However, tendon tissue overloading or underloading can induce remodeling toward a pathological state,^4^, ^5^ which is referred to as “tendinopathy.” Tendinopathy is characterized by drastic structural changes: anisotropy in the ECM is lost,^3^, ^6^ the ratio of collagen I to collagen III decreases while proteoglycan content increases,^6^, ^7^, ^8^ mechanical properties are compromised,^4^ vascular and neuronal ingrowth potentially cause pain,^2^ and cell numbers are increased, whereas cell alignment and elongated morphology are lost.^7^ The concomitant shift of the predominantly tenogenic cell population toward other mesenchymal lineages^8^, ^9^, ^10^, ^11^ decreases the functional remodeling capacity of tendon and aggravates the disorder.^12^, ^13^

Tendinopathy accounts for 30%‐50% of sports‐related injuries^5^ and nearly 30% of musculoskeletal issues‐related medical visits.^14^ Aside from physiotherapy and analgesic treatment, tendon disorders are hardly treatable due to gaps in knowledge concerning tissue healing and remodeling.^1^ Tendon culture model systems such as tissue explants—with a native ECM as key tissue feature—and in vitro cells, are widely used due to their practicality and to gain a better understanding of the fundamental disease progression and healing mechanisms.^15^, ^16^

However, culture systems using tendon‐derived cells (TDCs) with a reduced and therefore more controllable cell environment^15^ require cell isolation and expansion to minimize ethical concerns by maximizing cell yield per animal or to overcome the limited availability of human tissue for research. Expansion requires culture conditions showing similarities with tendinopathic tissue: hypercellularity, disorganized cells with loss of elongated morphology in the culture flask,^7^ and serum‐rich medium resembling neovascularization.^17^ Under these conditions, tenogenic cell populations lose their original healthy phenotype (“phenotypic drift”), via either transdifferentiation of cells or disproportional proliferation of subpopulations, similar to tendinopathic scenarios. Phenotypic drift likely increases by simplification of the model system, that is, extracting tissue from the body and further extraction of the cells from their native environment. As a result, phenotypic drift compromises translatability of tendon cell properties to in vivo tissue and hinders the development of effective treatment strategies.^18^, ^19^, ^20^

Limiting phenotypic drift of tendon cells in culture has been tried before^15^ by varying biophysical (eg, impose cell morphology using anisotropic substrates^12^, ^21^, ^22^, ^23^, ^24^, ^25^ or mechanical loading^20^), biochemical (eg, partial oxygen pressure or temperature^17^), and biological factors (eg, hormones, growth factors or cytokines^26^), while the precise combination, dosage and time‐dependent administration of various tenogenic stimulators remains to be determined. Nonetheless, blood serum, which provides a complex, poorly defined mixture of bioactive molecules, is widely used for in vitro TDC culture to increase cell proliferation and activity. This however, conflicts with the quiescent nature of healthy tendon cells,^1^, ^27^ and tendon‐specific phenotype seems to be maintained in low rather than high serum concentrations.^18^, ^26^ However, the effect of serum‐supplementation, tissue integrity cues (cell‐matrix contacts) and elongated cell morphology on the differential cellular response, and phenotypic drift of ECM‐embedded tendon cells and expanded TDCs has to our knowledge never been systematically quantified with respect to native tissue.^19^ Examining the potential of these factors to reverse phenotypic drift in TDCs is key to increase the physiological relevance of tendon culture models.

We, therefore, investigated phenotypic drift in tendon cells away from freshly isolated tissue and cells by separately controlling for complexity of the ECM niche and tendinopathic features of the microenvironment (serum and cell morphology). First, we used transcriptome analysis to screen for tendon‐relevant genes and quantify culture‐driven phenotypic drift in whole tendon explants—with a complex and intact ECM—upon serum‐free and serum‐rich culture. Subsequently, we validated serum‐dependent phenotypic drift in expanded TDCs and tested the potential for reversibility, by culturing TDCs in serum‐free or serum‐rich medium on random and aligned collagen I substrates—with a limited ECM complexity and controlled cell morphology. Their phenotypic gene expression was assessed and compared to freshly isolated TDCs. Hereby we highlighted the influence of the culture environment on the dynamic tendon cell phenotype and contributed to the increased physiological relevance of different in vitro model systems.

## 2 METHODS

### 2.1 Harvesting and culture of tendon fascicles

**Figure 1:**
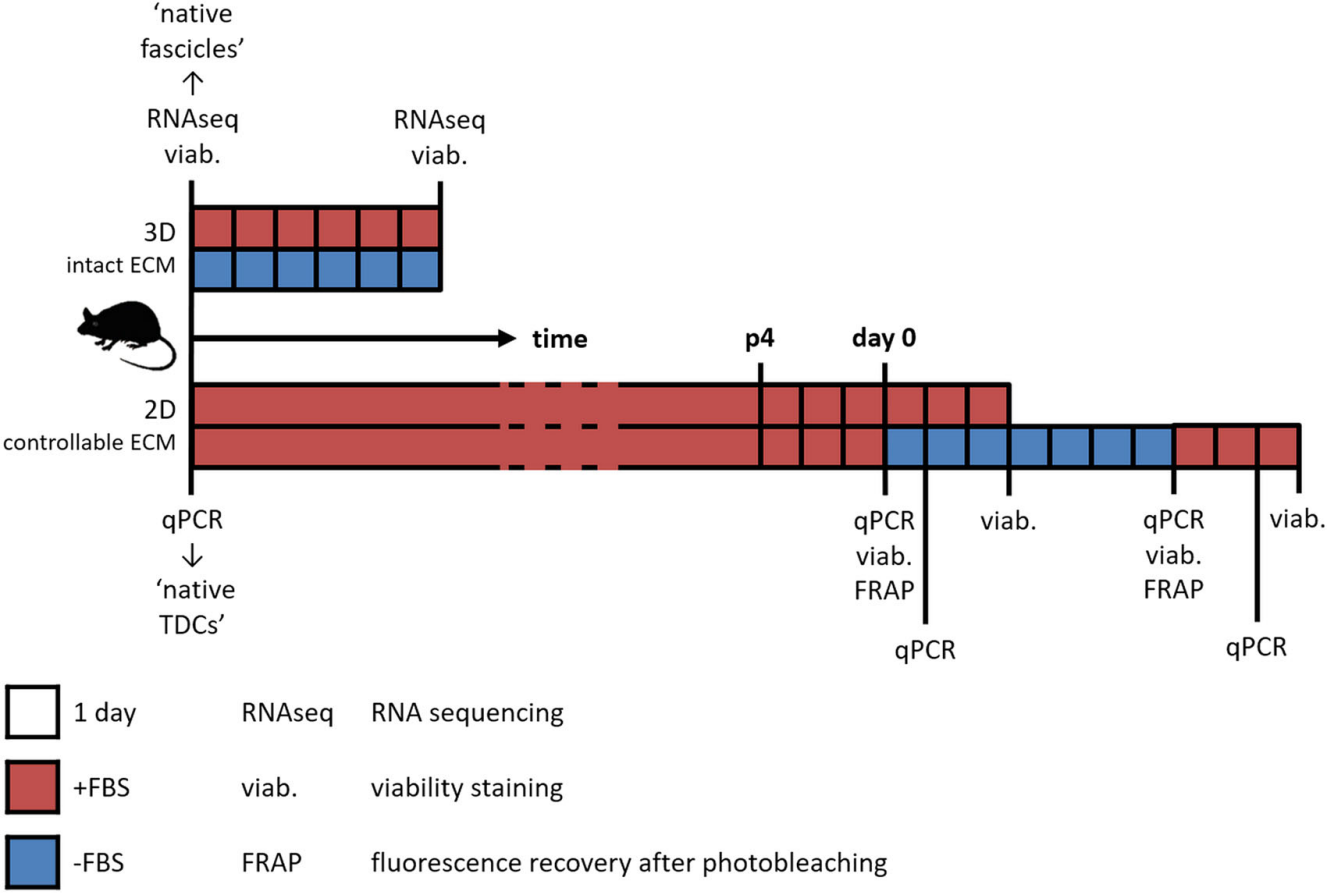
Schematic overview of experimental setup. Complete fascicles were cultured for 6 days in serum‐rich (+FBS) or serum‐free (−FBS) medium. mRNA was sequenced, cell viability was determined, and both readouts were compared to freshly isolated fascicles (“native fascicles”). TDCs were expanded in +FBS medium, and at sub‐confluency in passage 4 (day 0) medium was switched to +FBS or −FBS medium. Gene expression, cell viability, and functional intercellular communication were monitored over time, and gene expression was compared to TDCs directly after fascicle digestion (“native TDCs”). 2D, two‐dimensional; ECM, extracellular matrix; FBS, fetal bovine serum; mRNA, messenger RNA; qPCR, quantitative polymerase chain reaction; TDCs, tendon‐derived cells [Color figure can be viewed at wileyonlinelibrary.com]

All experiments on tendon fascicle explants were ethically approved by the Cantonal Veterinary office of Zurich (permit number ZH265/14). In total, nine C57BL6/J mice, 11 to 12‐week‐old, were euthanized and tail tendon fascicles were isolated as described before.^12^ Briefly, the tail tip was clamped with locking forceps and bent. The tendon fascicles were pulled out, hydrated in phosphate‐buffered saline (PBS) and cut off. Freshly isolated fascicles served as native control immediately after extraction. Explanted fascicles were cultured deprived from load at standard cell culture conditions (37°C, 5% CO_2_) in high‐glucose Dulbecco's Modified Eagle's Medium (HG‐DMEM; Sigma D6429) with or without 10% fetal bovine serum (FBS) (Gibco 26140079) for 6 days (Figure 1), as previously described.^17^

### 2.2 RNA sequencing and data analysis

RNA sequencing and data analysis were performed as previously described.^17^ In short, approximately 20 fascicles per animal (representing n = 1) were snap‐frozen in liquid nitrogen and pulverized in QIAzol lysis reagent by cryogenic grinding (FreezerMill 6870, SPEX SamplePrep). Tissue lysates were phase‐separated using 5Prime PhaseLock Gel Heavy (Quantabio). RNA was extracted by using the RNeasy micro Kit (Qiagen 74004), according to the manufacturer's instructions. The TruSeq Stranded mRNA Sample Prep Kit (Illumina, Inc, San Diego, CA) was used in the succeeding steps. Briefly, total RNA samples (100 ng) were poly‐A selected and then reverse‐transcribed into double‐stranded complementary DNA (cDNA), which was fragmented, end‐repaired, and adenylated before ligation of TruSeq adapters. The adapters contained the index for multiplexing. Fragments containing TruSeq adapters on both ends were selectively enriched with polymerase chain reaction (PCR). The libraries were normalized and the TruSeq SR Cluster Kit v4‐cBot‐HS (Illumina, Inc) was used for cluster generation using 8 pM of pooled normalized libraries on the cBOT. Sequencing was performed on the Illumina HiSeq 4000 single end 125 bp using the TruSeq SBS Kit v4‐HS (Illumina, Inc). Bioinformatic analysis was performed using the R package ezRun^28^ within the data analysis framework SUSHI.^29^ Raw read counts were normalized using the quantile method, and differential expression analysis was performed using the DESeq2 package.^30^ Only significantly and differentially expressed (upregulated/downregulated) genes between a serum condition and native were considered and were defined at the cut‐off values of |log_2_(fold change)| > 1 and *P* < .05 (Figure 3C). Exclusively differentially expressed genes met these requirements in one experimental group and not in the other. Subsets of significantly and differentially expressed genes were functionally enriched within the gene ontology domain “Biological process” using the Kyoto Encyclopedia of Genes and Genomes (KEGG) database.

### 2.3 Harvesting and culture of TDCs

TDCs were harvested from the tails of thirteen 7 to 9‐week‐old C57BL/6J mice that were euthanized as untreated control for other, unrelated experimental studies. When all tissues, as described in the respective permits, had been harvested, the tails were collected, and tendon fascicles were isolated as described above. The ECM was dissolved using sterile‐filtered 3 mg/mL collagenase IV (Gibco 17104019) in PBS for 4 hours at 37°C. TDCs were either lysed for gene expression analysis directly after tissue digestion as freshly isolated TDCs controls (pooled from two mice) or resuspended and expanded in complete growth medium, consisting of HG‐DMEM (Gibco 42430‐025) supplemented with 10% FBS (Greiner Bio‐One 758073/Bovogen SFBS), 1% penicillin‐streptomycin (Lonza DE17‐602E), and 1% nonessential amino acids (Gibco 11140‐035). Resuspended TDCs were seeded at 10 000 cells/cm^2^ in culture flasks coated with 50 µg/mL collagen I (Corning 354236) in PBS for 2 hours. The cells were cultured under standard conditions and passaged 1:3 every 3 to 4 days. Experimental readouts were performed at passage 4 (p4): TDCs were seeded on two‐dimensional (2D) substrates and grown to sub‐confluency in complete growth medium (referred to as day 0; Figure 1). At day 0, the culture medium was switched to complete growth medium, with or without 10% FBS for 7 days. To differentiate the effect of serum‐free medium from culture duration while preventing over‐confluency when using FBS, cells were not consecutively cultured in serum‐rich medium for 7 days, but medium was re‐supplemented with FBS for 2  days after 7 days serum deprivation (Figure 1).

### 2.4 Microcontact printing

**Figure 2:**
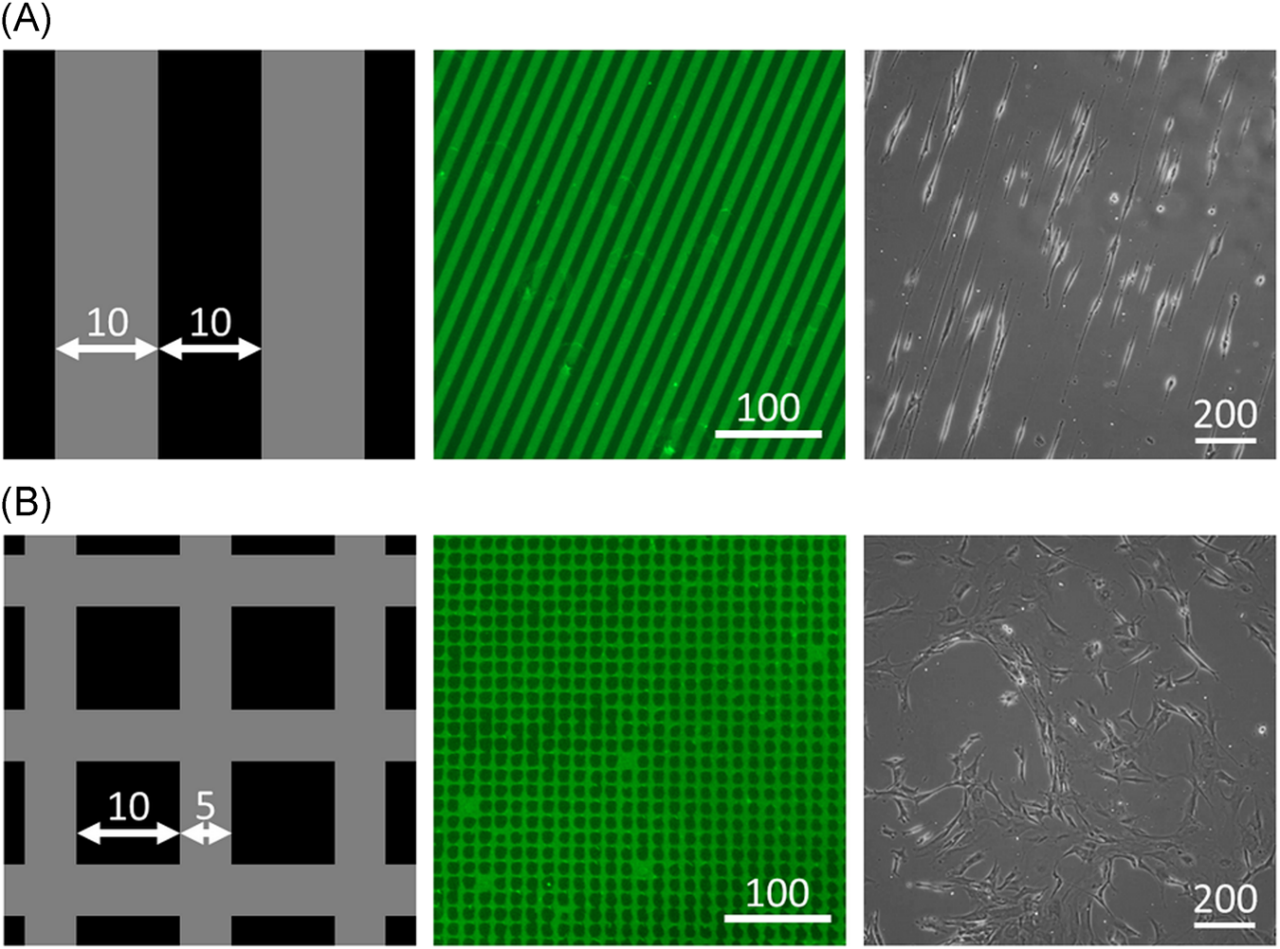
TDC morphology and orientation were controlled using microcontact printed collagen I patterns. Schematic of aligned (A) and random (B) microcontact printing patterns (1), with collagen I printed surfaces in gray and Pluronic‐F127–coated surfaces in black. Microcontact printed substrates with collagen I fluorescently stained in green (2) and resulting phase contrast microscopy images of seeded TDCs (3). All sizes are given in micrometers. TDCs, tendon‐derived cells [Color figure can be viewed at wileyonlinelibrary.com]

Collagen I patterns for controlling cell morphology and orientation were created using microcontact printing as described before.^31^ Briefly, 15 × 15 mm polydimethylsiloxane (PDMS; Dow Corning Sylgard 184) stamps were made, with either 10 µm wide lines and 10 µm spacing, or a “fishing net” structure with 5 µm wide lines and 10 µm spacing (Figure 2A1‐B1). These corresponded to aligned and random cell morphologies, respectively, with similar collagen I‐covered surface areas for cell attachment in both patterns (±50%).

Substrates were prepared by spin‐coating PDMS on coverslips, and curing overnight at 65°C. In all, 50 µg/ml collagen I in PBS was adsorbed onto the stamps for 1 hour, and meanwhile the substrates were hydrophilized with UV‐Ozone for 8 minutes. The stamps were blow‐dried with compressed air, gently pressed onto the substrates, and collagen was transferred for 15 minutes. Stamps were removed, substrates were washed 3× with PBS, and nonprinted areas were blocked with 10 mg/mL Pluronic‐F127 (Sigma P2443) in PBS for 5 minutes. Finally, the substrates were washed for 5 minutes with PBS and stored in PBS at 4°C until further use.

### 2.5 Quantitative polymerase chain reaction

TDCs (pooled from two mice) seeded on the microcontact printed patterns were grown to sub‐confluency in complete medium for 3 days. This moment of sub‐confluency is referred to as day 0 after which the culture medium was switched to serum‐free or serum‐rich HG‐DMEM or low‐glucose DMEM (Gibco 22320‐022). TDCs were lysed in RLT buffer directly after tissue digestion (“native TDCs”) and at days 0, 1, 7, or 9 (Figure 1), pooling up to four substrates to one sample. Total RNA was isolated using the RNeasy mini Kit (Qiagen 74106) and cDNA was synthesized using Moloney Murine Leukemia Virus reverse transcriptase (Invitrogen 28025‐013). Quantitative polymerase chain reaction (qPCR) primer sequences were obtained from literature or Primer‐BLAST,^32^ checked for specificity using Primer‐BLAST, and for proper efficiency using positive control mRNA dilution series. Sequences of primer sets (Sigma‐Aldrich) that passed all tests are listed in Table 1, and corresponding details are given in Supporting Information Table S1.

**Table 1:** qPCR primer sequences

Marker	Gene	F/R	Primer (5′ to >3′)
Tenogenic	Tenomodulin (Tnmd)	F	GCGATAATGTGACCATGTACTG
		R	GTCTTCTCCACCTTCACTTGC
	Scleraxis (Scx)	F	GTTGAGCAAAGACCGTGACAG
		R	CCGTGACTCTTCAGTGGCAT
	Tenascin C (Tnc)	F	TATCTGGTGCTGAACGGACTG
		R	CGGTTCAGCTTCTGTGGTAG
Tendon ECM	Collagen I, α1 (Col1a1)	F	AGCACGTCTGGTTTGGAGAG
		R	GACATTAGGCGCAGGAAGGT
	Collagen III, α1 (Col3a1)	F	CACGTAAGCACTGGTGGACA
		R	AGAAGTCTGAGGAATGCCAGC
	Decorin (Dcn)	F	GAGGGAACTCCACTTGGACAAC
		R	CCAGCTCGGCAGAAGTCATT
Myogenic	α‐Smooth muscle actin (Acta2)	F	GTGATCACCATTGGAAACGAAC
		R	GCATAGAGATCCTTCCTGATGTC
Fibrogenic	Connective tissue growth factor (Ctgf)	F	CAAGGACCGCACAGCAGTTG
		R	AGAACAGGCGCTCCACTCTG
Osteogenic	Alkaline phosphatase (Alpl)	F	GCAATGAGGTCACATCCATC
		R	CTCTGGTGGCATCTCGTTATC
Adipogenic	Fatty acid‐binding protein 4 (Fabp4)	F	GATGAAATCACCGCAGACGAC
		R	CCAGCTTGTCACCATCTCGTT
Housekeeping gene	Ribosomal protein L4 (Rpl4)	F	CTTCGCCAGGCCAGAAATCA
		R	TCTCGGATTTGGTTGCCAGTG
	Ribosomal protein S29 (Rps29)	F	CACGGTCTGATCCGCAAATACG
		R	GCATGATCGGTTCCACTTGGTA

Abbreviations: ECM, extracellular matrix; F, forward; qPCR, quantitative polymerase chain reaction; R, reverse.

For gene expression analysis, cDNA samples were diluted 60× in ddH_2_O, and cDNA amplification was measured in the CFX 384 Thermal Cycler (BioRad) for 40 cycles, using corresponding iQ SYBR Green Supermix (BioRad 1708886). *C*_t_‐values were normalized to the mean *C*_t_‐values of housekeeping genes Rpl4 and Rps29 and native TDCs (Figure 1).

### 2.6 Cell viability

Cell viability was assessed from fascicles of six different mice as previously described,^17^ and in adherent TDCs (pooled from nine mice). A detailed description of both methods is provided in the Supporting Information.

### 2.7 Fluorescence recovery after photobleaching

Intercellular communication was examined on randomly oriented TDCs (pooled from two mice) using fluorescence recovery after photobleaching (FRAP).^33^ Details are provided in the Supporting Information.

## 3 RESULTS

### 3.1 Phenotypic drift of cells in tendon explants is more pronounced in serum‐rich medium

To evaluate the impact of serum on the phenotypic drift of tendon cells within their native ECM, murine tail tendon explants were cultured in serum‐free or serum‐rich medium. Differential gene expression of tendon explants was assessed between native, freshly isolated, and cultured tail fascicles (Figure 1).

**Figure 3:**
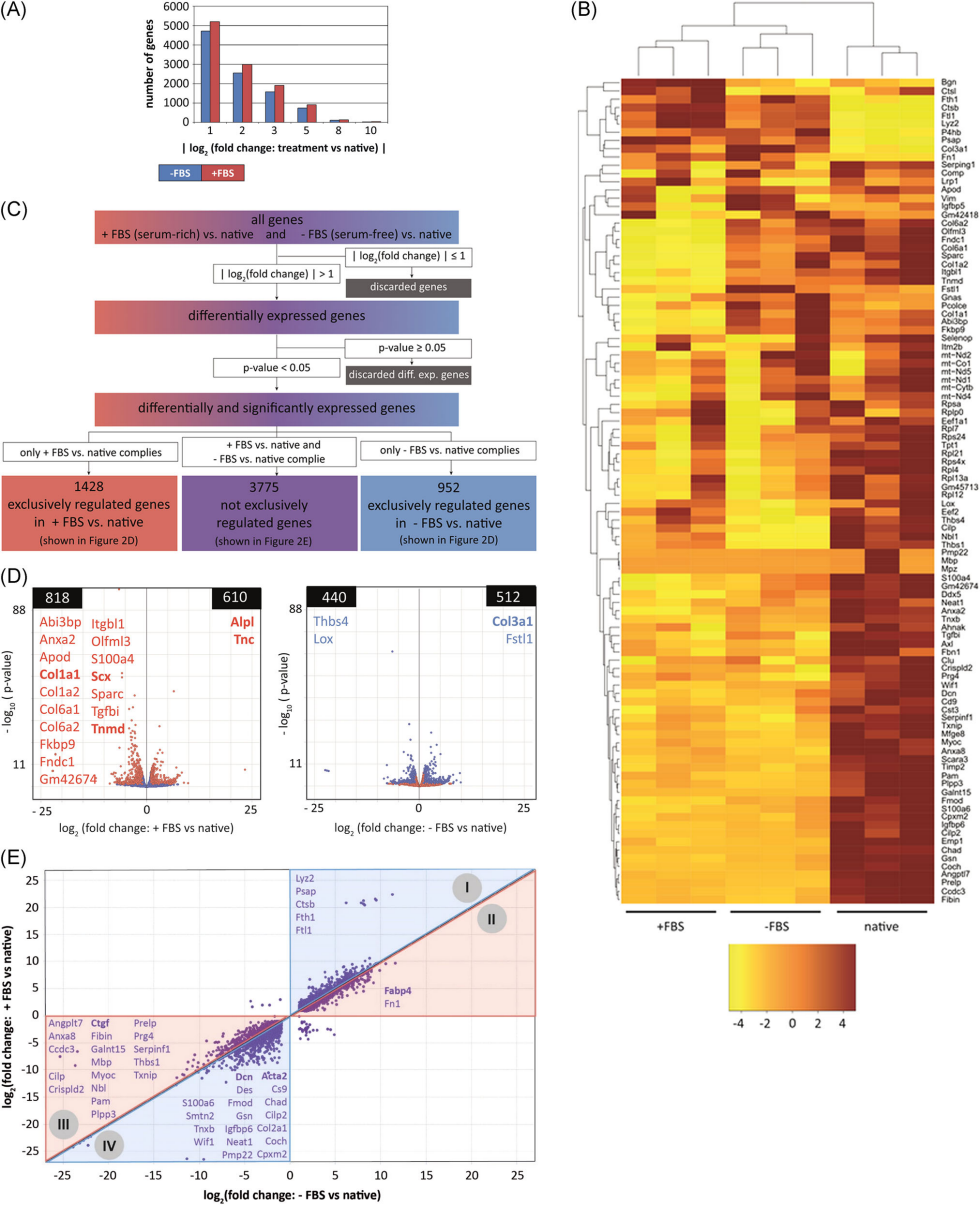
Serum induced phenotypic drift of tenocytes in tendon explants. RNA sequencing of native and explanted murine tail tendon fascicles cultured in serum‐rich (+FBS) or serum‐free (−FBS) medium. A, Number of significantly regulated genes (*P* < .05) between explant cultures and native tendon fascicles at various fold change cut‐off values. B, Heatmap of the 100 most highly expressed genes in native tendon according to the highest average normalized raw read counts. Depicted are normalized raw read counts of native fascicles and explants, cultured in serum‐rich or serum‐free medium. The heatmap is arranged using unsupervised hierarchical clustering in which columns and rows are grouped by similarity. Raw read count values of each gene (row) are separated by color using *z*‐score transformation, where positive and negative row‐scaled *z*‐scores are represented in brown and yellow, respectively. The *z*‐score indicates how many standard deviations the raw read count deviates from the mean of all the raw read counts in a row. C, Grouping of significantly and differentially regulated genes according to the selection criteria |log_2_(fold change)| > 1 and *P* < .05. D, Volcano plots showing the number of exclusively regulated genes between +FBS and native (red) or −FBS and native (blue) with the *P*‐value plotted against the |log_2_(fold change)| of +FBS vs native (right) or |log_2_(fold change)| of −FBS vs native (left). E, A total of 3775 significantly regulated genes in both conditions (+FBS and −FBS*)* when compared to native tissue (nonexclusive, displayed in purple in (C)). Triangles I and II show genes that are upregulated compared to native, whereas triangles III and IV are downregulated when compared to native. Genes with a blue background (triangles I and IV) benefit from serum‐free conditions by being regulated more toward native levels, when compared to serum‐rich conditions. Genes with a red background (triangles II and III) are regulated more toward native levels due to the serum‐rich conditions, compared to the serum‐free conditions. The diagonal black line represents genes that are equally regulated in +FBS and −FBS conditions, compared to native. Indicated gene abbreviations in (D) and (E) are all upregulated and downregulated genes of the top 100 most highly expressed genes in native tendon, as shown in (B). Genes that were investigated in the follow‐up cell culture experiments (see next paragraph) are highlighted in bold. n = 3, each n represents an independent pool of 20 fascicles from one mouse. FBS, fetal bovine serum [Color figure can be viewed at wileyonlinelibrary.com]

The number of differentially expressed genes compared to native was higher in tendon fascicles cultured with serum than in the serum‐free condition at all cut‐off values (Figure 3A). Based on expression of the 100 most active genes in native tendon tissue (Supporting Information Table S2), drastic phenotypic drift was observed in both the serum‐free and serum‐rich condition (Figure 3B). Strikingly, most of the tendon‐specific markers (Tnmd, Scx, Col1a1, Col1a2) were downregulated exclusively in the serum‐rich condition (Figure 3D and Supporting Information Table S3). This suggests a strong phenotypic drift away from the tenogenic lineage due to the serum‐rich environment. Genes upregulated exclusively in the serum‐rich condition comprised several vasculature‐ (eg, Wnt7b, Hif1a), immune system‐ and inflammatory‐associated markers (eg, Nfkb2, Il1a, Il20, Cxcl1, Hmgb2, Mmp1) and interestingly also the tendon marker Tenascin C (Tnc) (Supporting Information Table S3). Gene ontology analysis of these genes further indicated activation of processes related to proliferation, angiogenesis, inflammation, and immune response (Supporting Information Table S4). Genes regulated exclusively in the serum‐free condition included ECM (modulating) proteins (eg, Col3a1, Col6a5, Acan, Lox, Postn) and growth factors (eg, Tgfa, Fgf2) (Supporting Information Table S5) involved in different biosynthetic processes (Supporting Information Table S6). Overall, 1428 genes were significantly regulated, exclusively in the serum‐rich condition and 952 genes exclusively in the serum‐free condition, compared to native, but the majority of the genes significantly regulated relative to native (3775) were not exclusive, and thus in both the serum‐rich and serum‐free conditions. However, genes deviating less from native in serum‐free compared to serum‐rich conditions comprised tendon‐specific (eg, Dcn, Fmod) and several inflammation‐associated (eg, multiple Mmps, Il1b, Il11, Ccl2, Cxcl12) genes (Figure 3E and Supporting Information Table S7).

Cells showed comparable viability in native fascicles and serum‐free conditions, whereas cell number and viability were increased after 6 days of culture in the serum‐rich group (Supporting Information Figure S1A).

In summary, tendon tissue explant culture drastically changes cellular phenotype, and a serum‐rich environment exacerbates this phenotypic drift.

### 3.2 Serum deprivation for 7 days nearly restores native levels of phenotype marker genes in TDCs

We next aimed at assessing the phenotypic drift in cultured TDCs compared to native TDCs (lysed directly after tissue digestion) when cells were decoupled from their native ECM (Figure 1). Therefore, expression of phenotypic marker genes found to be relevant in the explant cultures was quantified in 2D expanded TDCs, in response to up to 9 days culture on random substrates in serum‐rich or serum‐free medium. Comparison of normalized read counts of native tendon fascicles and Δ*C*_t_ values of native TDCs showed strong correlation (*R* ^2^ = 0.91), indicating that cell isolation from the fascicle alone did not drastically affect gene expression in TDCs (Supporting Information Figure S2).

**Figure 4:**
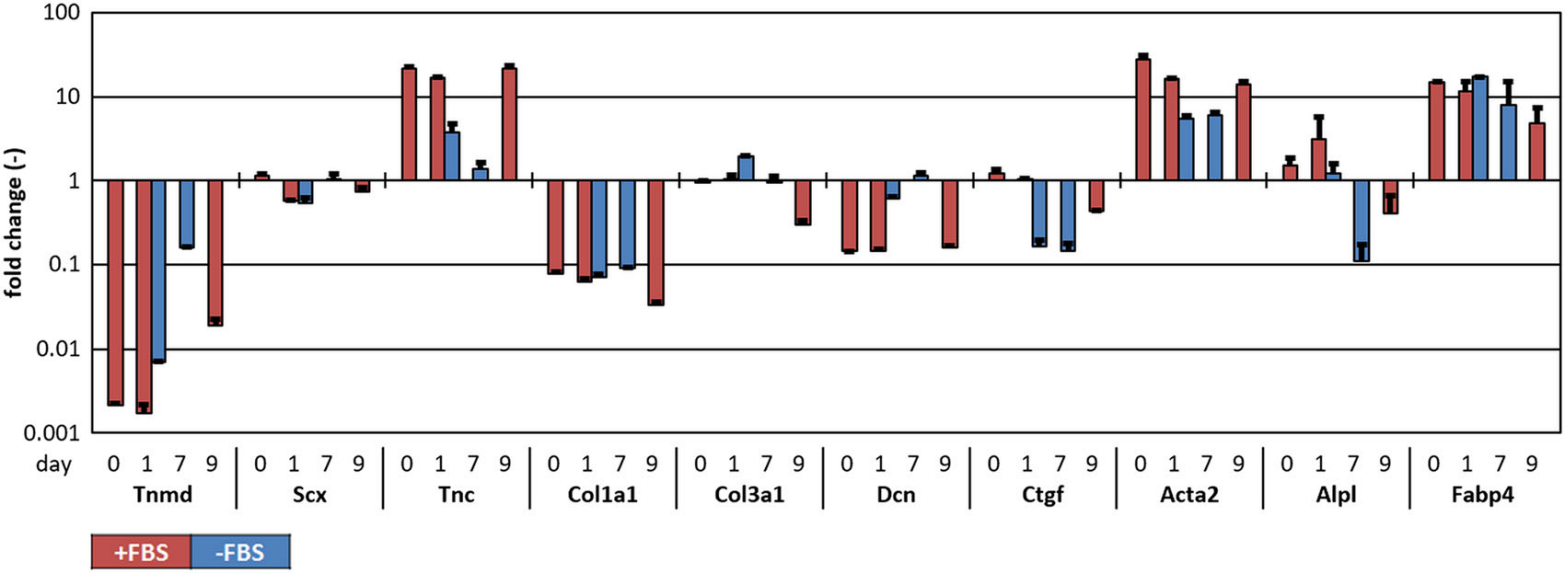
Gene expression of expanded TDCs (p4) cultured in serum‐rich and serum‐free medium on random substrates for 7 days after the moment of sub‐confluency (day 0), compared to freshly isolated TDCs (fold change = 1). After 24 h, serum deprivation had a “rescuing” effect on phenotype marker genes (Tnmd, Tnc, Dcn, Acta2) in TDCs that were expanded in serum‐rich medium. Tendon‐specific phenotypic markers were almost restored to native levels in p4 TDCs after 7 days of serum deprivation, and this effect was counteracted by serum supplementation for 2 days. n = 2. TDCs, tendon‐derived cells [Color figure can be viewed at wileyonlinelibrary.com]

Strong phenotypic drift was observed in the expanded TDCs in serum‐rich medium, which was indicated by an impressive deviation from native gene expression levels (fold change = 1) for Tnmd, Tnc, Col1a1, Dcn, Acta2, and Fabp4 at day 0 (Figure 4). All of the above‐mentioned genes were regulated toward native levels in serum‐free medium after 1 day of culture except for Col1a1 and Fabp4, which remained similar to serum‐rich conditions. Only Ctgf and Col3a1 diverged from native levels without serum, while remaining at original levels in serum‐rich medium. Upon 7 days in serum‐free medium, Acta2 and Col1a1 remained stable compared to day 1. Scx varied over time, but did not show a clear serum‐dependency. Tnmd, Tnc, and Fabp4 continued to converge toward native levels, whereas the fold changes for osteogenic marker Alpl dropped below 1 (Figure 4 and Supporting Information Figure S3). Remarkably, Tnmd gene expression increased almost 100‐fold upon 7 days serum deprivation.

Strikingly, gene expression levels of Tnc, Dcn, and Acta2 nearly approached levels of day 0 cells within 2 days after switching the medium back to serum‐rich in serum‐deprived samples. In the same time span, Tnmd dropped 10‐fold, compared to day 7 (Figure 4).

### 3.3 The effects of morphology and glucose level on TDC phenotype are negligible compared to serum concentration

To assess whether confining TDCs to an elongated morphology further rescues gene expression toward native levels, murine TDCs were seeded on random and aligned microcontact printed collagen I substrates. Figure 2A3‐B3 confirms that TDCs seeded on these substrates adapted their morphology to the patterns. All changes in gene expression were shown to be independent of cell orientation (Supporting Information Figure S4).

To rule out any gene modulatory effect of the high glucose level in the culture medium, gene expression levels of in vitro TDCs were assessed in high‐ and low‐glucose DMEM. No phenotypic recovery in low glucose levels was detected (Supporting Information Figure S4).

### 3.4 Serum deprivation slightly affects cell viability and does not affect intercellular communication

TDC viability in serum‐rich and serum‐free conditions was assessed. Number and percentage of live cells dropped approximately 25% upon 7 days serum deprivation, resulting in a similar distribution as in native and serum‐deprived fascicles. Remarkably, many cells died when serum was added after 7 days serum deprivation (Supporting Information Figure S1B).

To rule out differences in cell connectivity, we examined gap junction functionality—an important functional hallmark of tenocytes—by FRAP. We compared TDCs expanded in serum‐rich medium (day 0) and after 7 days of serum deprivation on random substrates. Serum deprivation did not abolish fluorescence recovery in serum‐deprived TDCs, indicating that functional intercellular communication was maintained (Supporting Information Figure S5).

Altogether, in these 2D experiments, serum‐rich medium induced strong deviations from native gene expression levels, whereas 7 days of serum deprivation appeared to reverse this phenotypic drift, particularly for the tenogenic marker and tendon ECM genes (as classified in Table 1). No large adverse effects on TDC viability or functionality were found. The impact of cell morphology and glucose level appeared negligible in this system (Table 2)

**Table 2:** Expression and significance of selected tendon‐specific markers for explanted fascicles in the +FBS and −FBS culture condition

Gene	+FBS		−FBS	
	*P*‐value	log_2_(fold change)	*P*‐value	log_2_(fold change)
Biglycan (Bgn)	.001	0.57	.90	−0.02
Collagen I, α1 (Col1a1)	6.35 × 10^−18^	−3.10	.11	0.61
Collagen I, α2 (Col1a2)	3.32 × 10^−22^	−3.40	.86	0.07
Decorin (Dcn)	2.61 × 10^−12^	−1.76	8.03 × 10^−5^	−1.13
Mohawk homeobox (Mkx)	.004	−0.91	.72	−0.10
S100 calcium binding protein (S100a4)	4.49 × 10^−10^	−1.51	.002	−0.79
Scerlaxis (Scx)	3.03 × 10^−14^	−3.53	.01	−0.81
Tenascin C (Tnc)	.01	1.25	.36	1.04
Tenomodulin (Tnmd)	7.45 × 10^−30^	−4.16	.01	−0.82
Thrombospondin 4 (Thb4)	.11	−0.48	9.34 × 10^−16^	−1.95

Abbreviation: FBS, fetal bovine serum.

## 4 DISCUSSION

The relevance of in vitro tendon culture models is compromised by phenotypic drift of tendon‐derived cells, in response to high serum levels and random cell morphology.^18^, ^21^, ^22^, ^23^ While previously phenotypic properties were compared between cultured cells in different passages^19^, ^21^ or various culture conditions within the same passage number,^18^, ^22^, ^23^, ^25^ quantification and comparison of phenotypic drift in tendon culture models with respect to native tissue and TDCs was scarcely described.^34^ In this study, we explored the phenotypic drift of tendon cells by stepwise reduction of model system complexity using first explanted tendon fascicles with preserved native three‐dimensional (3D) ECM and then expanded TDCs on 2D substrates. Using these model systems, we investigated whether native cell microenvironment of tendon explants, serum deprivation, or elongated in vitro TDC morphology could limit the loss and stimulate the restoration of tenogenic phenotype in tendon cell culture systems.

Transcriptome analysis of tendon fascicle explants cultured in serum‐rich vs serum‐free medium—mimicking pathologically vascularized vs limitedly nourished healthy tissue, respectively—showed that tissue explant culturing downregulated the most highly expressed tendon genes, and that serum‐rich medium aggravated this phenotypic drift. Serum supplementation directed tendon explant cells to an active, inflammatory phenotype, whereas serum‐deprivation stimulated biosynthetic processes. This might explain the difficulties in resolving tendinopathy, that is, soluble blood factors increase after local damage, directing cells in healthy areas toward inflammation and thereby possibly aggravating tendinopathy.

Next, using microcontact printed collagen I patterns—providing the most prominent substrate molecule in healthy tendon tissue, but without an out‐of‐plane structural component—we decoupled the influence of native cell‐matrix contacts from the bioactive effect of serum and matched the purely 2D structure of regular expansion cultures. TDCs experienced a significant phenotypic drift upon in vitro expansion in serum‐rich medium similar to—but stronger than—the explant model (eg, Tnmd: 0.002‐fold vs 0.05‐fold). This indicates that intact tissue architecture tends to alleviate phenotypic drift. Serum deprivation revealed a highly dynamic serum‐responsive nature of TDCs, which is independent of cell morphology and glucose concentration: tendon‐specific genes (Tnmd, Dcn, Tnc) converged or even fully recovered toward native levels upon serum‐deprivation, while subsequent serum supplementation reversed gene expression changes induced by serum deprivation. Serum deprivation slightly decreased cell viability—remarkably more closely resembling native fascicles than serum‐supplemented cultures.

In both culture models (explants or in vitro TDCs) native gene expression levels were highly similar, and all measured genes showed the same differential gene expression patterns for the serum‐free and serum‐rich conditions compared to the native controls, except for Scx, Acta2, Col1a1, and Fabp4. This indicates that cues from serum‐derived factors override matrix‐integrity cues for these genes. The fascicle explant is expected to provide lower substrate stiffness than in vivo tendon, whereas the thin PDMS film on glass in vitro is expected to be stiffer. Proposedly, this relates to the mechanosensitive nature of certain differentially regulated genes in the explant vs the TDC culture, like Scx,^35^ which was downregulated in the free‐floating fascicle and constantly expressed in vitro. Similarly, Acta2 equilibrates cytoskeletal tension to substrate stiffness,^36^ and was decreased in the load‐deprived, lower substrate stiffness explant culture. The constant, serum‐independent, Col1a1 expression in vitro may be ascribed to the absence of ascorbic acid in the culture medium, which is essential for collagen transcription.^37^

Technical feasibility of the experiments and ethical concerns required expansion of TDCs until passage 4. During this cultivation period certain cellular subpopulations were possibly selected, which may additionally explain differences between the 2D and 3D model systems. Besides that, the collagen coating of the substrates in all passages may have influenced proliferation of certain TDC subpopulations.^38^, ^39^ Collagen I coating has been shown to promote TDC adhesion, proliferation,^40^ and elongated morphology,^41^ and the ability of the TDCs to adhere to collagen was essential due to the performed cell patterning method with collagen substrates. Moreover, we cannot conclusively ascribe the observed phenotypic dynamics to more proliferative cellular subpopulations or transdifferentiation. Considering the phenotypic shift observed after a relatively short culture period of 1 day in a serum‐free (nonproliferative) environment, we speculate that transdifferentiation is the driving mechanism.

Despite abundant literature describing the negative influence of FBS or some of its components (eg, bone morphogenetic proteins, transforming growth factor‐β, platelet‐derived growth factor, fibroblast growth factor) on tenocyte‐like phenotype,^18^ the underlying mechanism has not been elucidated. There is however reason to believe that xenogenic serum may result in an unnatural cell behavior and protein release pattern,^42^ possibly by differences in epigenetic patterns and resultant differentiation potential of stem cells according to the use of autologous or xenogenic serum.^43^ However, for practical and financial reasons FBS is currently the standard in cell culture experiments, making our culture conditions a proxy for the numerous tendon cell culture experiments that use FBS. It remains to be shown that the use of autologous serum is more beneficial for tenogenic phenotype expression.

We speculate that high serum levels in vitro, resembling neovascularization in vivo, may induce a switch in tenocytes toward a more active, metabolic, inflammatory phenotype (supported by our ontology analysis), analogous to tendinopathy, and similar to cells that crossed the “metabolic tipping point,” which recruit extrinsic tissue compartments to heal the damaged tissue.^1^ Strikingly, Tnmd—the gene that was most affected by serum—has an anti‐angiogenic role in (healthy) tendons.^44^ This implies a positive feedback loop of low tissue vascularization promoting Tnmd expression, which in turn prevents angiogenesis. On the other hand, Tnc might be a pro‐angiogenic factor, which was increased in the vascularized (serum‐rich) model and is similarly found in blood vessel‐infiltrated tendon tissue.^3^

The serum‐induced phenotypic drift during in vitro cell expansion appears to be reversible by serum deprivation. This is a simple and effective procedure to obtain a large number of tenocyte‐like cells for in vitro studies, compared to mechanical, topological, biological, or biochemical stimuli that are currently applied to induce tenogenic differentiation of stem cells or maintaining phenotype in primary TDC expansion.^15^, ^26^

Previously, cell alignment has been shown to increase tenogenic markers (eg, Tnmd: 80‐ to 200‐fold) during stem cell differentiation,^22^, ^23^, ^45^ although this effect was not detected consistently.^24^ However, the increase in tenogenic markers upon cell alignment in primary TDCs rarely exceeds 5‐ to 10‐fold,^21^, ^25^, ^45^ similar to our results.

The options and boundaries of serum deprivation as experimental phenotypic drift‐limiting method need to be further determined. Firstly, prominent tendon markers Col1a1 and Tnmd did not completely recover to native levels in this research, but expression of these genes can potentially be stimulated biochemically with ascorbic acid or additional bioactive stimuli.^46^ Secondly, all in vitro readouts in this research were performed at p4. However, phenotypic drift may also be reversible at higher passages, which would be ethically favorable due to a maximized cell yield per animal, reducing the number of animals sacrificed for in vitro tendon research. Thirdly, with qPCR and RNA sequencing, the most important readouts in this research focused on gene expression levels. These are indicators—but no direct measures—for protein production, cell behavior, and phenotype.^47^ However, “alterations in (…) gene expression” are explicitly mentioned in the definition of phenotypic drift,^15^ justifying the choice for these readouts. It is important to consider that gene expression analysis by transcriptomics and qPCR requires a certain level of mRNA production to exceed the detection limits. Serum starvation decreases the production of mRNA^27^ and therefore requires high numbers of cells in order to guarantee feasibility of the readout. The clinical implications need to be explored as well. Despite the fact that rodent animal models of (tendon) tissue repair can deviate from human system behaviors,^48^ for instance in immune system involvement during healing,^49^ an impressive phenotypic plasticity is observed in both murine and human TDCs.^50^ This arguably gives confidence for the clinical translatability of the presented findings in the search for an established tendinopathy treatment. Improving properties of tendinopathic cells and tissues by controlling vascularization —analogous to serum deprivation in this research promoting a healthier tenocyte‐like phenotype—might be a potential path to follow in the quest for a clinical tendinopathy treatment aiming at (partial) tissue recovery.

In summary, this study quantified the phenotypic drift away from native gene expression in different tendon culture models revealing an extremely dynamic phenotype of tendon cells. It provided a valuable set of tendon maker genes that contributed to the further characterization of the underresearched tendon tissue. We conclude that serum supplementation exacerbates phenotypic drift in tendon tissue explant and in vitro cell culture systems, and overrides cues from ECM integrity, cell orientation, and morphology. Serum deprivation limits phenotypic drift in cells of explanted tendon tissues, reverses it after in vitro expansion, and therefore represents a method to potentially increase physiological relevance of in vitro studies.

## AUTHOR CONTRIBUTIONS

MvV and SLW conceived and designed the analysis, collected the data, and performed the analysis. MvV, SLW, and JF wrote the paper. KI, JGS, and JF supervised the project. All authors provided critical feedback and helped shape the research, analysis, and manuscript. All authors have read and approved the final submitted manuscript.

## Supporting information

Supplementary information

Supplementary information

Supplementary information

Supplementary information

Supplementary information

Supplementary information

Supplementary information

Supplementary information

Supplementary information

Supplementary information

Supplementary information

Supplementary information

Supplementary information

## Data Availability

The RNA‐sequencing data from this publication have been deposited to the Annotare database (https://www.ebi.ac.uk/fg/annotare) and assigned the identifier E‐MTAB‐9540.
