# Supplementary figures and images for: Serum deprivation limits loss and promotes recovery of tenogenic phenotype in tendon cell culture systems
Source: J Orthop Res. 2020 Jun 10;39(7):1561–71. doi: 10.1002/jor.24761 (PMC8359397; doi:10.1002/jor.24761)

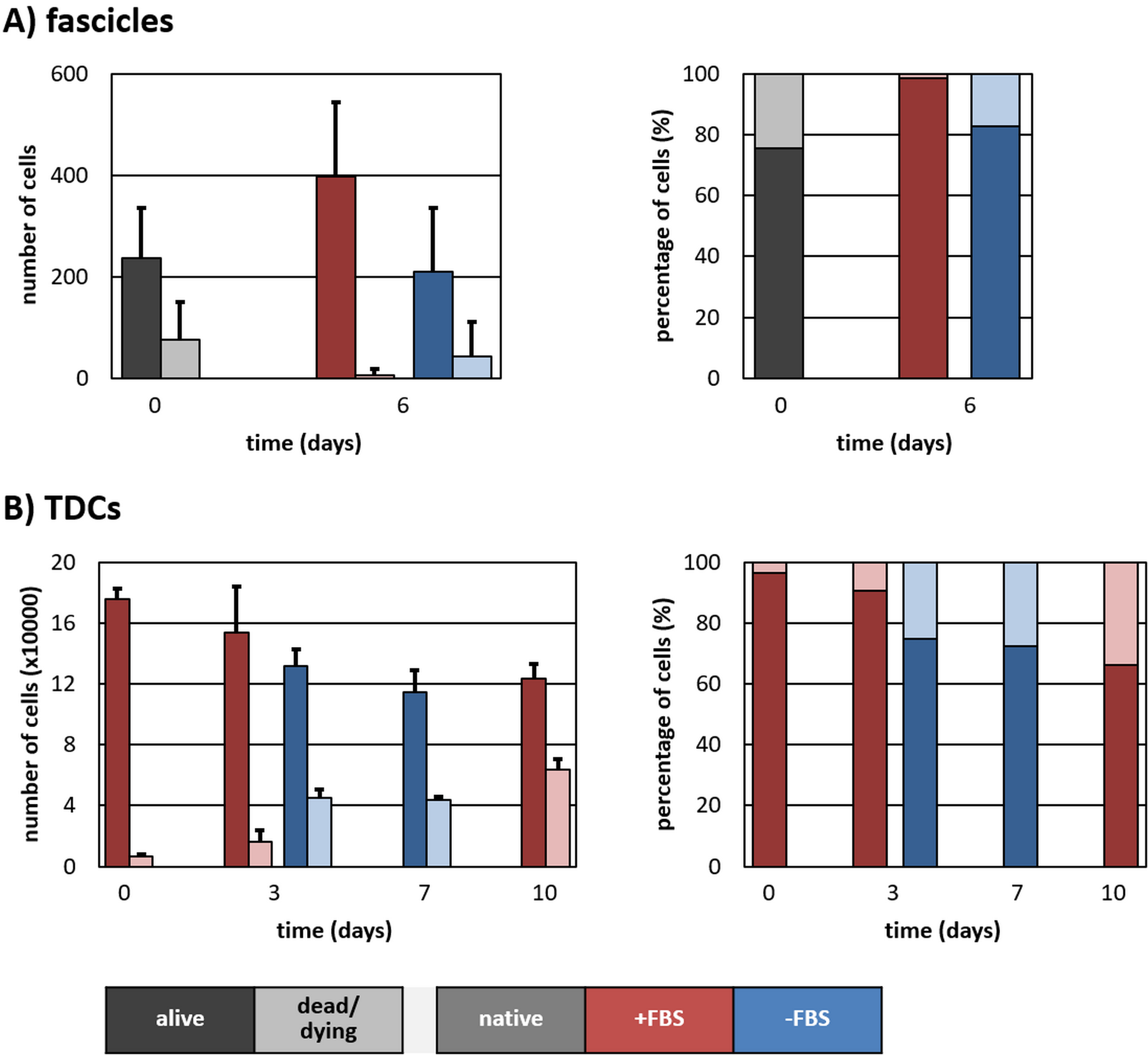

Supplement: Supplementary file 1 — Supplementary information [file JOR-39-1561-s012.tif]

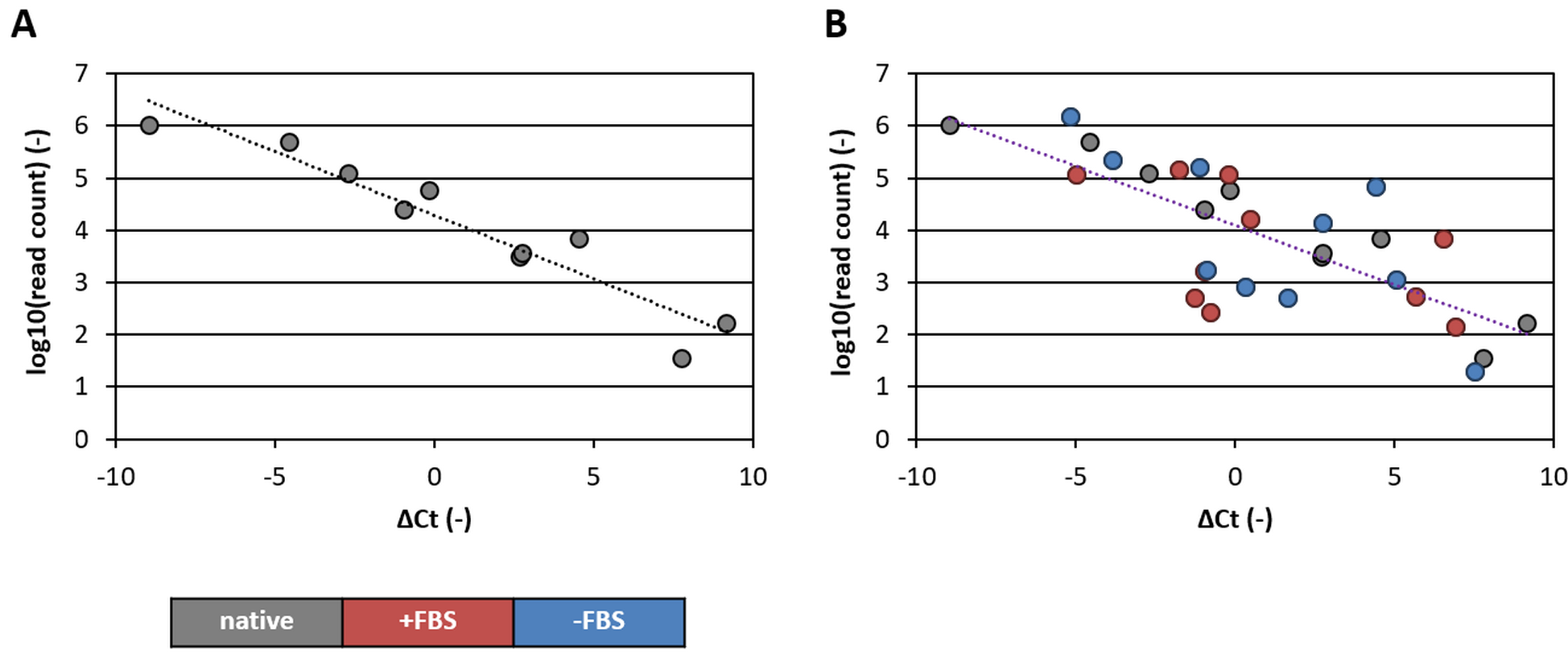

Supplement: Supplementary file 2 — Supplementary information [file JOR-39-1561-s006.tif]

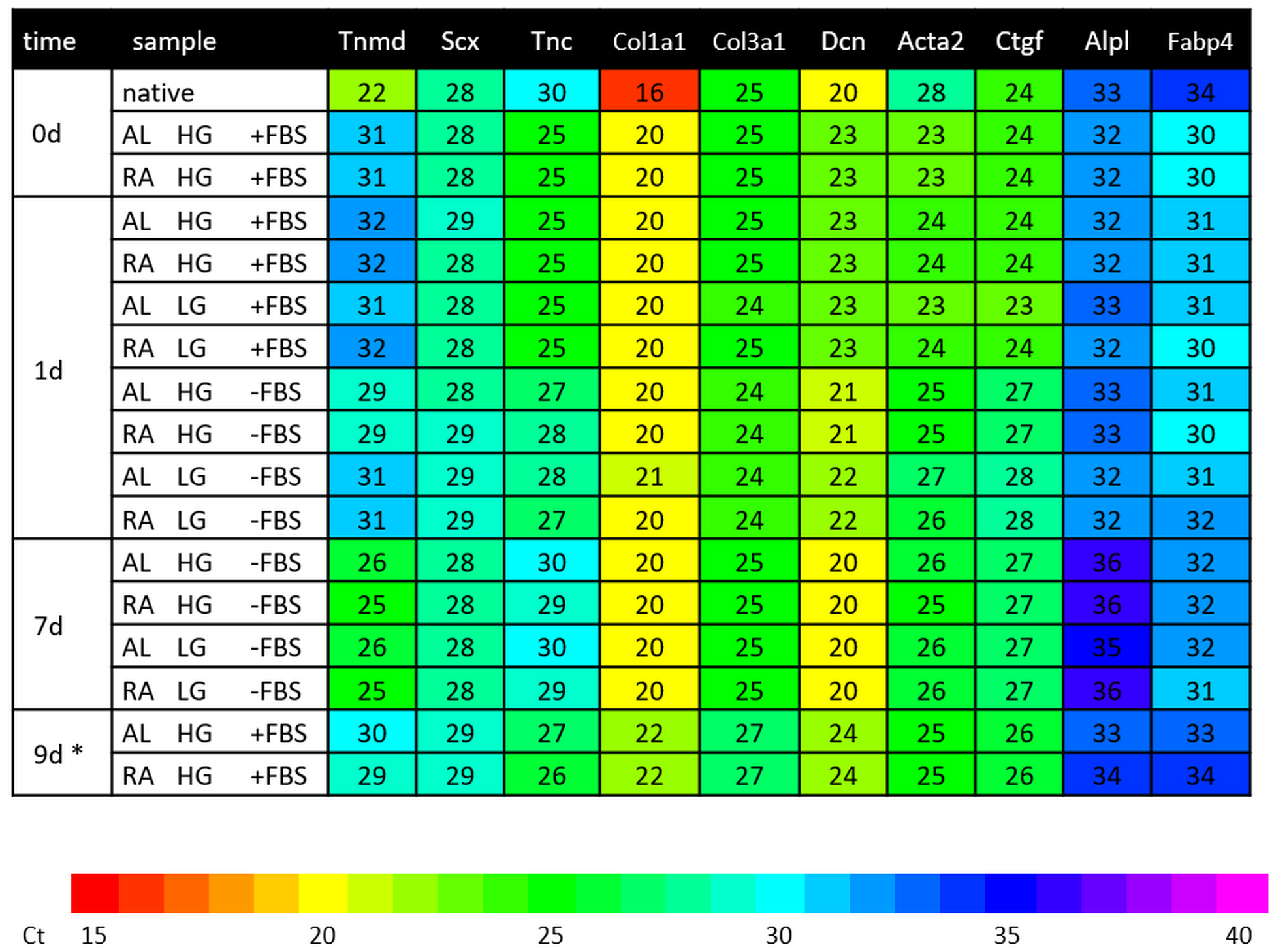

Supplement: Supplementary file 3 — Supplementary information [file JOR-39-1561-s001.tif]

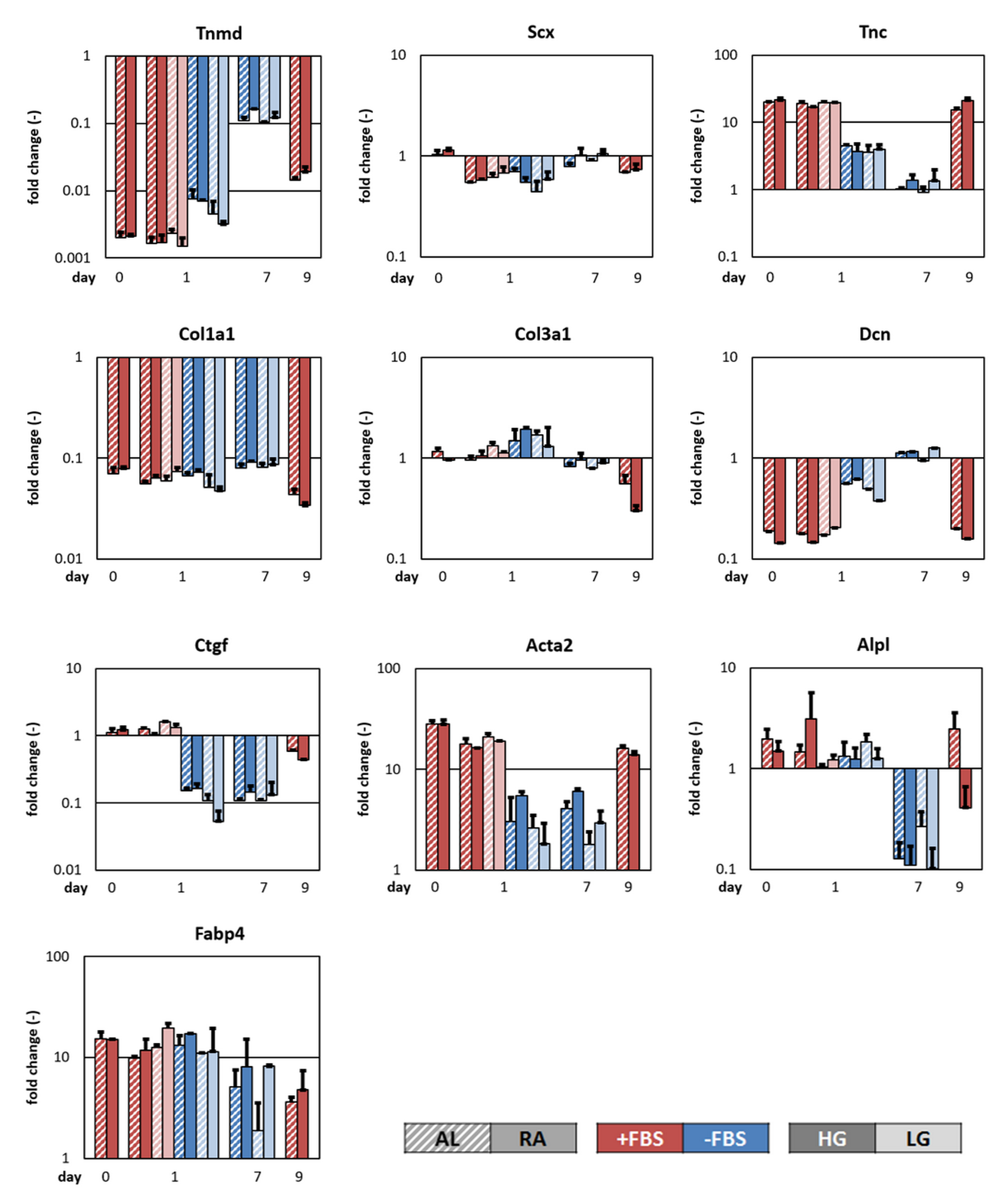

Supplement: Supplementary file 4 — Supplementary information [file JOR-39-1561-s004.tif]

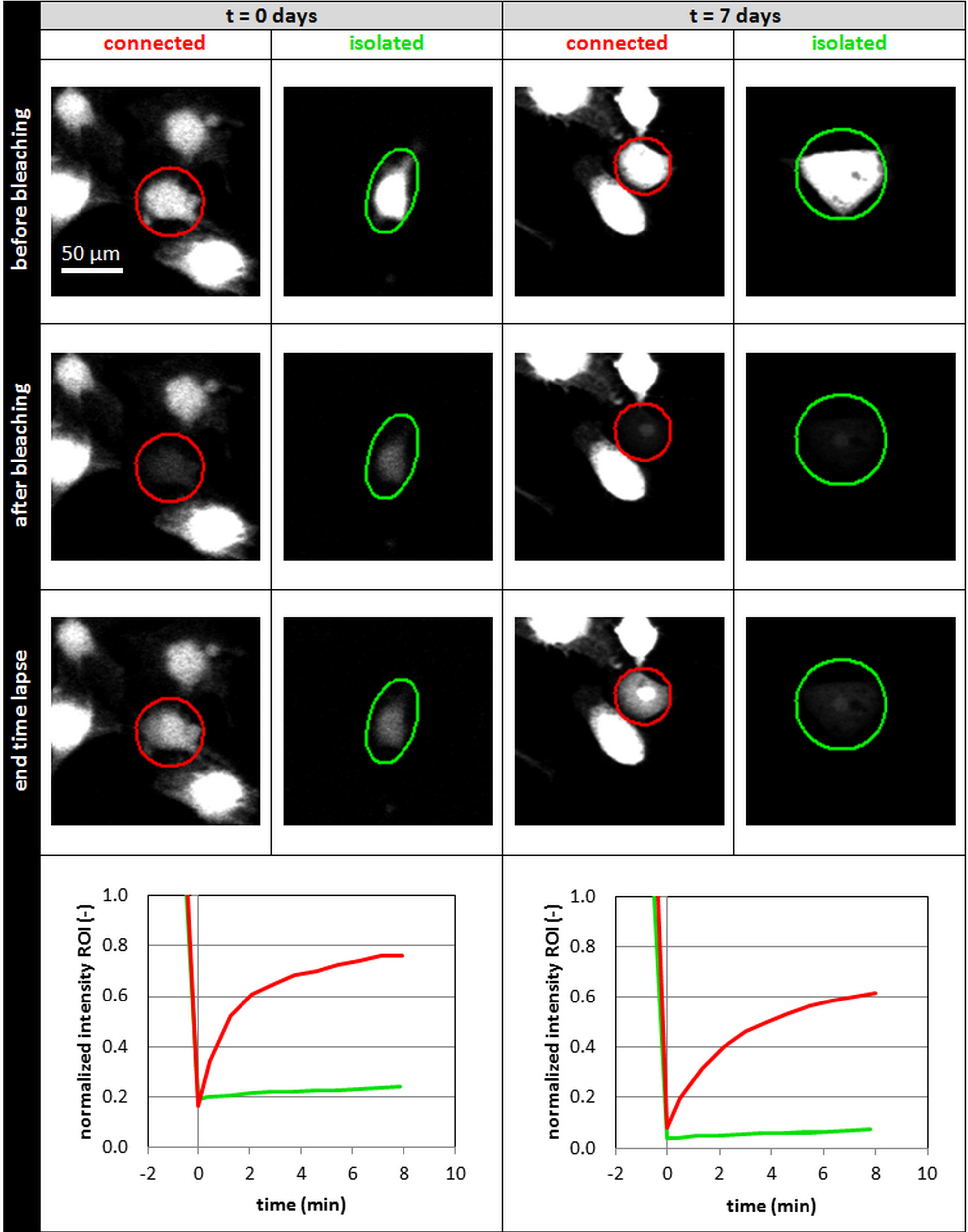

Supplement: Supplementary file 5 — Supplementary information [file JOR-39-1561-s010.tif]
