# Supplementary material for: Serum deprivation limits loss and promotes recovery of tenogenic phenotype in tendon cell culture systems
Source: J Orthop Res. 2020 Jun 10;39(7):1561–71. doi: 10.1002/jor.24761 (PMC8359397; doi:10.1002/jor.24761)
Supplement: Supplementary file 13 — Supplementary information [file JOR-39-1561-s011.docx]

**Supplementary methods**

***Cell viability fascicles***

Cell viability was assessed from independent fascicles of six different mice directly after tissue extraction or after 6 days of explant culture. Single fascicles were fixed in 10% formalin (Sigma Aldrich, HT5011) for 20 minutes. Dead/dying cells in the fascicles were stained with Ethidium Homodimer-1 (Anaspec AS83208) and cell nuclei were stained with NucBlue reagent (ThermoFischer R37606). Whole fascicles were embedded in a 50% (v/v) glycerol (Sigma-Aldrich, G5516) solution in PBS. Z-stack fluorescent images were obtained in triplicates by using a spinning disc confocal microscope (iMic, FEI). Dead/dying and live cells (total cell number minus dead/dying cells) and percentage live cells (live cells divided by total cell number) were quantified by means of a custom microscopic image analysis tool in Matlab (MathWorks).

***Cell viability in TDCs***

Viable and adherent dead/dying cells were assessed in adherent TDCs (pooled from nine mice) at day 0, 3, 7 and 10. Additionally, non-adherent dead cells were counted in the collected medium of adherent TDC cultures using a haemocytometer. Adherent TDCs were washed with PBS and subsequently stained with Hoechst 33342 (5 μg/ml, Invitrogen H3570) and Ethidium Bromide (20 μg/ml, Sigma-Aldrich E1510) simultaneously for 20 min, staining all cell nuclei and adherent dead/dying cells, respectively. Three complete wells (9.6cm^2^ per well) with stained cells per condition were imaged with a Leica DMi8 microscope, and quantified using ImageJ (National Institutes of Health). Percentage live cells was calculated as described for cell viability in fascicles.

***Fluorescence recovery after photobleaching (FRAP)***

Collagen I patterns were microcontact printed in glass bottom dishes (Thermo Scientific™ Nunc™ 15235672). TDCs were washed with PBS, incubated with 1 µg/ml calcein acetoxymethyl (Sigma 17783) in PBS for 20 min at 37°C, and washed with PBS. FRAP was performed on a Zeiss LSM Meta NLO confocal microscope (Argon laser excitation at 488 nm, emission filter BP 500-530 nm) by creating circular or elliptical regions of interest (ROIs) covering single cells. Selected TDCs connected to neighboring cells were evaluated for intercellular communication capacity (recovery of the bleached signal), whereas isolated cells in the same cultures served as negative controls. A baseline scan was followed by 300 bleaching iterations at 50% laser power, a scan immediately after bleaching, and ten more scans every 50 s. The average ROI intensity was determined and normalized to baseline using a custom-made script in Matlab.

**Supplementary figures**

***Figure S-1:*** *Cell number and viability in (A) tendon fascicles and (B) TDCs. Cell number and viability are generally higher for cells cultured in serum-rich medium (+FBS), whereas viability of cell cultured in serum-free medium (-FBS) resembles freshly isolated fascicles (native) better.*

***Figure S-2:*** *Comparison of log_10_(read count) and ΔCt values for all genes of interest in fascicle transcriptome analysis and TDC qPCR, respectively. (A) Gene expression in freshly isolated fascicles and TDCs (native) highly correlated (R^2^ = 0.91). (B) Including gene expression in fascicles (day 6) and TDCs (day 1) cultured in serum-rich or serum-free medium this correlation decreased (R^2^ = 0.57), but still similar patterns were observed for both culture systems.*

***Figure S-3:*** *Ct values for the investigated genes. Absolute gene expression levels of phenotype marker genes for TDCs cultured on aligned (AL) or random (RA) substrates, in high glucose (HG) or low glucose (LG) DMEM, with (+) or without (-) FBS, compared to native levels. * 9d culture comprised 7-day serum deprivation followed by 2-day serum supplementation.*

***Figure S-4:*** *Gene expression of TDCs cultured in serum-rich and serum-free medium on random and aligned substrates in high or low glucose medium. Serum deprivation had a larger (positive) impact on phenotype marker genes in p4 TDCs than low glucose level or cell alignment, and phenotypic markers were almost restored to native levels upon 7 days of serum deprivation. This effect was counteracted immediately when serum-rich medium was added. Relative gene expression levels of phenotype marker genes compared to native levels (fold change = 1) for TDCs cultured to sub-confluency (0d), and afterwards for 1 day or 7 days on aligned (AL) or random (RA) substrates, in high-glucose (HG) or low-glucose (LG) DMEM, with (+) or without (-) FBS. Medium with FBS was added to samples after 7 days serum deprivation, and these were cultured for 2 more days.*

***Figure S-5****: Fluorescent recovery of TDCs after photobleaching. Serum deprivation for 7 days did not abolish intercellular communication. FRAP for randomly oriented TDCs cultured in serum-rich medium (t = 0 days) and after serum deprivation (t = 7 days). Microscopy images from before and directly after bleaching, and at the end of the time lapse. The bottom row shows the quantification of average fluorescence intensities in ROI normalized to baseline. For each condition, a minimum of 4 different cells were scanned, and representative series are shown.*
